# A DECADE OF STROKE CAREGIVER RESEARCH: THE RESCUE CREST STUDY

**DOI:** 10.1093/geroni/igz038.2494

**Published:** 2019-11-08

**Authors:** Constance R Uphold, I Magaly Freytes, LeLaurin Jennifer, Schmitzberger Magda, Nathaniel Eliazar-Macke, Tatiana Orozco, S Angelina Klanchar, Stuti Dang

**Affiliations:** 1 North Florida/South Georgia Veterans Health System, Gainesville, Florida, United States; 2 North Florida/South Georgia Veterans Health System, Gainesville, United States; 3 James A. Haley Veterans’ Hospital, Tampa, Florida, United States; 4 Miami VA Healthcare System, Miami VA Healthcare System, Florida, United States

## Abstract

Stroke caregivers need education and support to care for suddenly disabled family members. To address this need, we are conducting a nurse-led intervention, called RESCUE CREST, to teach caregivers problem-solving skills using an educational website and an online messaging center. In our first, preliminary study, we pre-tested an investigator-created, senior-friendly website called RESCUE (Resources and Education for Stroke Caregivers’ Understanding and Empowerment) using focus groups and interviews with clinicians and caregivers. We refined the website based on additional surveys, a cognitive usability study, and Webtrends analysis. In the second study, we used a single-group pre- and post-test design to evaluate the content and outcomes of the problem-solving intervention. Third, we conducted a four-arm RCT (4-week intervention, 8-week intervention, attention control, standard care) to refine our methods. We will describe the lessons learned and results of our 10 years of stroke caregiver research that provide background for the RESCUE CREST study.

